# Revisiting ancient DNA insights into the human history of the Pacific Islands

**DOI:** 10.1002/arco.5180

**Published:** 2019-03-19

**Authors:** MATTHEW SPRIGGS, FREDERIQUE VALENTIN, STUART BEDFORD, RON PINHASI, PONTUS SKOGLUND, DAVID REICH, MARK LIPSON

**Affiliations:** The Australian National University and Vanuatu Cultural Centre; CNRS, France; The Australian National University and Vanuatu Cultural Centre; University of Vienna; Francis Crick Institute, London; Harvard Medical School, the Howard Hughes Medical Institute, and the Broad Institute of MIT and Harvard; Harvard Medical School

## Abstract

We respond to issues raised in the recent Forum on “Ancient DNA and its contribution to understanding the human history of the Pacific Islands” in AO (Bedford et al. 2018). We first present an emerging model for the early peopling of Vanuatu combining the genetic and archaeological evidence. Secondly, we respond specifically to the criticisms of two contributors: Matisoo-Smith and Sand. We discuss various misconceptions about the Teouma Lapita cemetery and about sampling issues in DNA research.

## INTRODUCTION

We were glad to see the serious attention to the recent studies of ancient DNA from Remote Oceania in the Forum in *Archaeology in Oceania* (Bedford et al. 2018, henceforth ‘Forum’). Most of the contributors were highly positive about the value of the studies. The contributors also raised important issues involved in contextualizing the genetic findings within archaeology and linguistics.

We begin by highlighting a philosophical point that guided our writing team in Lipson et al. (2018) and the earlier Skoglund et al. (2016). In both papers, we took the approach of foregrounding the genetic results, letting them speak for themselves rather than presenting them as part of an integrated argument with the archaeological and linguistic evidence. We agree with Kirch and other Forum contributors that archaeological and linguistic evidence are no less important than the genetic evidence, and that a combined consideration of all data is the end goal. However, a feature of such ‘triangulation’ when it is done well, as in Kirch and Green (2001), is the initial independence of the lines of evidence being compared.

In what follows, we first describe an integrated model for the early peopling of Vanuatu and then address criticisms raised by Matisoo-Smith and Sand in the Forum.

## TOWARDS A REVISED MODEL FOR THE PEOPLING OF VANUATU

We hypothesize that Near Oceanic-related ancestry (which we will henceforward call ‘Papuan’) first became widespread in Vanuatu in the Late Lapita period. By contrast, the initial Lapita migration stream (when the Vanuatu population was small) was comprised almost entirely of people of ‘First Remote Oceanian (FRO)’ ancestry, a term we introduced in Skoglund et al. (2016) to be able to discuss the East Asian-related ancestry observed in the Talasiu and Teouma individuals independent of terms used for language or material culture. Over time, a new stream of primarily Papuan ancestry largely replaced the first, FRO-ancestry stream, leapfrogging from the Bismarck Archipelago to the Reefs-Santa Cruz Islands and on to Vanuatu. The second stream involved little exchange with people from the main Solomon Islands (Sheppard 2011), consistent with the specific relationship of Papuan ancestry in both present-day and ancient Ni-Vanuatu to the Papuan ancestry in the Bismarck Archipelago found in both Lipson et al. (2018) and Posth et al. (2018).

Jointly considering the results of the two genetic studies, it may be that there were different dynamics for the initial arrival of Papuan-related ancestry in particular parts of the Vanuatu archipelago, although additional data are necessary to further explore this question. Such differences would be unsurprising in light of the archaeological and linguistic evidence of cultural heterogeneity across the archipelago (Bedford and Spriggs 2018). However, present-day groups have relatively homogeneous proportions and sources (ultimately from the Bismarck Archipelago) for their Papuan ancestry, plus similar reconstructed average dates of mixture between Papuan and FRO ancestry sources (albeit with some exceptions; Lipson et al. 2018). This supports the idea of a relatively discrete ‘second wave’ (Blust’s ‘M2’, Forum, p. 206). Had the pattern instead been a slow infiltration of diverse populations over many centuries, we would not expect such homogeneity, especially given that the nearest source area geographically is the Solomon Islands.

Archaeological evidence of material culture connections between the Bismarck Archipelago and Vanuatu broke down at the end of Lapita: never again do we observe transport of New Britain obsidian to Vanuatu, nor is there any convincing evidence showing direct connections between Post-Lapita pottery styles on either side of the Remote Oceania–Near Oceania boundary. Sand (Forum, p. 215) suggests that such contacts did continue in the immediate Post-Lapita period, but we are not aware of documentation of this theory from archaeology or genetics, and he provides no citation.

From the aDNA data, we now know that at c. 2900 cal. BP in Vanuatu (as well as from a culturally comparable Lapita context in Tonga at c. 2650 cal. BP), there were FRO-ancestry populations with genetic profiles very different from those of any Ni-Vanuatu today. Additionally, by 2500–2300 cal. BP, there were individuals with almost entirely Papuan ancestry in the centre (Efate) and south (Tanna) of the archipelago – again outside the range of ancestry proportions of present-day Ni-Vanuatu peoples. All three genetics papers agree that present-day Ni-Vanuatu are not simple descendants without mixture of the people who lived in the archipelago in its first 500 years of human occupation, and that the history of the region involves profound population transformations.

Of course, there are many gaps still to be filled; in Vanuatu, a particular priority for future study is the period from 2800–2500 cal. BP. It remains possible, as argued by Matisoo-Smith (Forum, p.211) that there could have been individuals with considerable amounts of Papuan ancestry in Vanuatu earlier than c. 2800 BP. However, even beyond the results discussed above, there are several pieces of evidence suggesting that Papuan impact occurred after this time. Both 2018 papers showed by analyzing later Vanuatu individuals that admixture between Papuan and FRO ancestry sources occurred predominantly in Late-Lapita times at the earliest. The only known individuals with almost entirely FRO ancestry in Vanuatu date to the earlier Lapita period, while the two individuals referenced above with entirely Papuan ancestry represent the earliest known appearance of such ancestry, as would be expected from relatively rapid and directed migrations followed by slow mixture between previously separated groups.

## RESPONSE TO CRITIQUES

While eight of the ten contributors were generally positive about the scholarly value of the genetic studies, two (Matisoo-Smith and Sand) were critical. In this section we address their most salient critiques.

### 1. Matisoo-Smith suggests that the Teouma skulls are unrepresentative of the ancestry of those bearing the Lapita culture in Remote Oceania.

The first important argument against this suggestion is the fact that three Lapita individuals from Tongatapu in Tonga with aDNA data have the same FRO ancestry profile as the Teouma Vanuatu individuals about 2000 kms to the west (Lipson et al. 2018; Posth et al. 2018; Skoglund et al. 2016). Our paper in 2016 already showed that the first people of Remote Oceania included a widely-distributed population who had extremely low proportions of Papuan-related ancestry – far less than the minimum of c. 25% found in Remote Oceania today. The three published aDNA papers are based on small sample sizes, but provide definitive insights by using those samples to contradict hypotheses that were previously dominant in the genetic literature, specifically the prevalent view “that the first people in Remote Oceania and Polynesia had substantial Papuan ancestry” (quote from Skoglund et al. 2016:510, citing previous studies by Kayser 2010, Wollstein et al. 2010 and Matisoo-Smith 2015).

Matisoo-Smith questions whether the Teouma cemetery skulls derive from people who were representative of the early Lapita period in Vanuatu. She stresses the “unusual presence and context” of these skulls, and speculates they were “perhaps an unusual subset or possibly curated skulls of ancestors or even offerings” (Forum, p. 211, 212), claiming that the preliminary report of Valentin et al. (2010) “point[s] out that it could not be determined if the skulls from Teouma came from the cemetery or from an entirely different location.” However, further studies published in the past 10 years have revealed close similarities between the crania and incomplete burials at the site, thus supporting the idea of their belonging to a single community.

Specifically, all adult individuals at Teouma were involved in a single mortuary ritual that involved skull manipulation. This was a complex burial treatment, involving a sequence of activities over time (Valentin et al. 2010, 2016). As part of this process, crania were removed from all adult burials after body decomposition, and some crania were re-used in funerary rituals to honour particular individuals or descent lines.

Further evidence has come from isotopic study of the individuals buried at Teouma. First, carbon and nitrogen isotopes from the bone collagen of 65 individuals, including the crania tested for aDNA (Petchey et al. 2014 and Kinaston 2010), places the large majority (seven crania and 47 incomplete burials) in the same range of variation. These individuals thus shared the same dietary habits, at least during the last 10 years of their lives, with food resources taken from a single local food web. Second, contrary to Matisoo-Smith’s suggestion (Forum, p. 212), strontium and oxygen isotopic study of dental enamel (Bentley al. 2007) has provided no evidence of a “non-local” origin for the individuals with crania at Teouma. Of 17 studied individuals (13 incomplete burials and four crania, B10A, B10B, B10C and B17, of which we have aDNA data for B10B and B17), four represented by incomplete burials spent their childhoods on coral islands, while the other 13 (nine incomplete burials and the four crania) formed a separate isotopic cluster, again supporting the similarity across burial type.

Matisoo-Smith argues that the strontium isotopic values for Teouma and other Pacific populations such as those in the Bismarck Archipelago are “remarkably similar” and that “[t]herefore based on the isotope data, the Teouma crania could have come from many other parts of the Pacific” (Forum, p. 212). However, this is irrelevant to the question of whether or not the Teouma skulls are representative of the Lapita culture-associated individuals of Efate; the stable isotope and radiocarbon data make it clear that they are typical. The parsimonious interpretation is that the skulls and the incomplete burials in the cemetery are from a single population. The individuals buried at Teouma have a clear association with the Lapita culture, and strong evidence of being from a population with no more than a few percent Papuan ancestry (confidence interval of 0.1–4.7% for the sampled individuals) (Lipson et al. 2018).

### 2. Matisoo-Smith suggests a contradiction between Skoglund et al. (2016) and Lipson et al. (2018) with respect to proportions of Papuan ancestry in the FRO population.

In Skoglund et al. (2016:510), we indeed wrote that FRO populations “had little to no Papuan ancestry” – our study reported confidence intervals of 0–11% for Vanuatu and 0–17% for Tonga. In Lipson et al. (2018), with further sequencing from the same individuals as well as an additional Teouma individual, we refined the estimate to 2.4% ± 0.9% (confidence interval of 0.1–4.7%: Lipson et al. 2018:1158). Thus, the most likely estimate of Papuan ancestry at Teouma actually *decreased* in the second study, although with broad overlap in the uncertainty ranges. The key and consistent observation is that Papuan ancestry was much smaller than in any Remote Oceanic population of the past 2500 years (all >25%).

### 3. Matisoo-Smith and Sand both question whether we can make strong conclusions with a limited number of samples.

Sand writes: “are less than a dozen samples from a handful of sites statistically meaningful to write anew the first phase of human settlement in Remote Oceania” (Forum, p.215)? However, this argument reveals a misunderstanding of the nature of genomic data. Unlike mitochondrial DNA and Y chromosome analyses, genome-wide studies of a single individual draw on information from thousands of that individual’s ancestors. Thus, the relevant sample size for genome-wide DNA data is not the number of individuals, but a composite of the number of individuals and the number of independent regions of the genome studied.

Even a single ancient genome can be definitive. For example, as noted above, one hypothesis for the present-day distributions of admixed ancestry in Remote Oceania has been that FRO- and Papuan-ancestry populations mixed in the vicinity of New Guinea, and (separate) admixed descendants of these people moved to Vanuatu and to Polynesia (with more and less Papuan ancestry, respectively) (e.g. Matisoo-Smith 2015). However, the documentation of early inhabitants of Vanuatu and Tonga with almost entirely one or the other type of ancestry shows that additional, more complex movements of people must have been involved in these processes and, in particular, reveals a widespread group with extremely low proportions of Papuan ancestry that is the only ancestry type observed to date in people living prior to c. 2500 cal. BP across two widely-separated archipelagoes in Remote Oceania.

### 4. Matisoo-Smith, Sand and some other contributors express concerns about how these genetic findings interact with the perspectives of indigenous communities.

This concern is expressed most thoughtfully by Burley, who, while positive about the scholarly findings, is concerned about how the discovery that present-day people of Vanuatu are not descendants without admixture of the first Lapita settlers of the islands could be misinterpreted. In particular, he is worried that the genetic results might be seen as contradicting previous public communication about there being a close link between early Remote Oceanian archaeological sites like Teouma and people today. He and Sand both highlight as an example the Kanaks and the effect that ancient DNA findings might have on discussions about who is truly indigenous in New Caledonia. The truth is that genetic studies have shown that all people in Remote Oceania have substantial proportions of FRO ancestry—a range of 10–75% (Skoglund et al. (2016)—derived from early bearers of the Lapita culture such as those buried at Teouma. Thus, Burley’s concern (Forum, p. 208) that the data are supporting the idea that “Polynesians are the ancestral [sic] vestige of Lapita peoples” and by implication other Remote Oceanians are not, in fact reflects a misunderstanding. As archaeology, genetics and linguistics show, in a very real sense all the indigenous people living in the Pacific Islands today are descendants of the first inhabitants of Remote Oceania.
